# Harnessing the health systems strengthening potential of quality improvement using realist evaluation: an example from southern Tanzania

**DOI:** 10.1093/heapol/czaa128

**Published:** 2020-11-06

**Authors:** Fatuma Manzi, Tanya Marchant, Claudia Hanson, Joanna Schellenberg, Elibariki Mkumbo, Mwanaidi Mlaguzi, Tara Tancred

**Affiliations:** 1 Health Systems, Impact Evaluation and Policy, Ifakara Health Institute, Dar es Salaam, Tanzania; 2 Department of Disease Control, London School of Hygiene and Tropical Medicine, UK; 3 Department of Public Health Sciences, Karolinska Institutet, Sweden

**Keywords:** Implementation research, realist evaluation, quality improvement, health systems strengthening, maternal and newborn health, LMICs

## Abstract

Quality improvement (QI) is a problem-solving approach in which stakeholders identify context-specific problems and create and implement strategies to address these. It is an approach that is increasingly used to support health system strengthening, which is widely promoted in Sub-Saharan Africa. However, few QI initiatives are sustained and implementation is poorly understood. Here, we propose realist evaluation to fill this gap, sharing an example from southern Tanzania. We use realist evaluation to generate insights around the mechanisms driving QI implementation. These insights can be harnessed to maximize capacity strengthening in QI and to support its operationalization, thus contributing to health systems strengthening. Realist evaluation begins by establishing an initial programme theory, which is presented here. We generated this through an elicitation approach, in which multiple sources (theoretical literature, a document review and previous project reports) were collated and analysed retroductively to generate hypotheses about how the QI intervention is expected to produce specific outcomes linked to implementation. These were organized by health systems building blocks to show how each block may be strengthened through QI processes. Our initial programme theory draws from empowerment theory and emphasizes the self-reinforcing nature of QI: the more it is implemented, the more improvements result, further empowering people to use it. We identified that opportunities that support skill- and confidence-strengthening are essential to optimizing QI, and thus, to maximizing health systems strengthening through QI. Realist evaluation can be used to generate rich implementation data for QI, showcasing how it can be supported in ‘real-world’ conditions for health systems strengthening.

**KEY MESSAGES**

- Quality improvement can be utilized to overcome health systems bottlenecks and contribute to health systems strengthening.
- How and why this occurs, and therefore, how it may be maximized, can be understood through realist evaluation.
- Realist evaluation can also generate a transferrable programme theory for quality improvement implementation that may be useful across contexts.

## Introduction

### Health systems strengthening and quality improvement

Health systems are the networks of people and institutions who exist primarily to promote, restore or maintain health (World Health Organization, 2007). Maternal and newborn health (MNH) outcomes are sometimes seen as proximal indicators of health systems functioning: throughout the course of pregnancy, childbirth and into the postpartum and newborn periods, women and their babies may receive care at the primary through tertiary level-facilities; these outcomes are influenced by social determinants of health; and changes in health system functioning can be shown to directly impact them (Walley *et al.*, 2008; Koblinsky *et al.*, 2016; Bhagavathula *et al.*, 2017). Maternal and newborn morbidity and mortality are concentrated in the poorest countries in the world, many of which are found in Sub-Saharan Africa (Alliance for Maternal and Newborn Health Improvement Mortality Study, 2018). Institutional delivery has long been hailed as an important means by which maternal and newborn deaths can be reduced (The Partnership for Maternal Newborn and Child Health, 2015). However, despite marked increases in institutional delivery across Sub-Saharan Africa, maternal and newborn morbidity and mortality still persist (Alkema *et al.*, 2016). This paradox reflects, among other things, the need to continue to improve the quality of care provided.

Quality improvement (QI) has no standard methodological definition (Walshe, 2009). There are a multitude of QI approaches, some of the best-known ones involve the use of plan-do-study-act (PDSA) or plan-do-check-act cycles (Petersen, 1999; Speroff and O'Connor, 2004), although certain types of audit and feedback are also frequently used, particularly in clinical settings (Weeks *et al.*, 2010; Pirkle *et al.*, 2011). Here, we are referring to the participatory approaches through which stakeholders engaged within a process identify context-specific problems and create and implement solutions to these. Local data are collected to determine whether improvements have been made, so the entire process is data-driven. If improvements are not observed, or are minimal, new or adapted solutions are implemented and measured in a cyclical process. QI can be used to overcome barriers to implementing proven packages of care in MNH, e.g. basic and comprehensive emergency obstetric care (Kongnyuy *et al.*, 2008; Afari *et al.*, 2014; Dickson *et al.*, 2015).

QI interventions have also been used for ‘diagonal’ health systems strengthening (Gounder and Chaisson, 2012; Mahomed *et al.*, 2014). That is, using one targeted area of improvement (e.g. MNH) to strengthen deficits across the health system. QI engages stakeholders from across the health system, builds problem-solving skills and strengthens different building blocks of the health system (Colbourn *et al*., 2013, Davies *et al*., 2008, Doherty *et al*., 2009, Leatherman *et al*., 2010). For example, improving the collection and use of local data may bolster information systems (Swanson *et al.*, 2010); engaging health facility staff in processes that empower them to exact control over their working conditions may improve staff retention, strengthening human health resources; and service delivery improves as a result of QI activities. Furthermore, these approaches have been hailed by healthcare leaders and improvement experts as particularly relevant in low-income settings, given the emphasis on locally designed solutions, which minimize the need for external resources; use of participatory approaches, which may be better taken up in community-orientated social systems; and finally, because gaps in quality may be more pronounced in low-income settings, which is when QI can be most effective (Smits *et al.*, 2002). As QI engages stakeholders from across the health system, users of health services can and have been incorporated into QI processes (Tesfaye *et al.*, 2014; Waiswa *et al.*, 2017; Tancred *et al*., 2018). This may be particularly relevant to MNH services, where there is renewed interest in patient experiences of care and use of patient insights in enhancing both the quality and people-centeredness of services (Bohren *et al.*, 2019).

### Learning from QI

Despite QI processes being numerous and commonly used to improve healthcare across Sub-Saharan Africa (Franco and Marquez, 2011; Peters *et al*., 2013; Heiby, 2014; Kringos *et al.*, 2015; Lee *et al.*, 2016; Wells *et al.*, 2018), there is a lack of robust evaluation of both the implementation and impact of QI interventions (Marshall *et al.*, 2013; Garcia-Elorrio *et al.*, 2019). While QI appears technically simple and is theoretically appropriate within the challenges of persistent resource constraints, QI initiatives often fail to embed or to sustain within health systems, which is compounded by a lack of insights around implementation processes (Smits *et al.*, 2002; Hulscher *et al.*, 2013; Nadeem *et al.*, 2013; Wells *et al.*, 2018). Furthermore, constraints around a lack of political will and inadequate buy-in from leaders, poor availability of resources, insufficient manpower and time among practitioners to utilise QI effectively and a lack of requisite skills—especially data literacy—to use QI have been highlighted as key barriers to QI implementation (Ingabire *et al.*, 2015; Ritchie *et al.*, 2016; Stokes *et al.*, 2016; Wagenaar *et al.*, 2017). Surmounting these requires insights about how they may have been overcome in settings with shared barriers. Heiby (2014) has called for emphasis on learning from and sharing QI experiences within and between African countries, and, critically, an expansion of organizational learning around the sustainability and spread of improved practice and institutionalization of quality. Such learning will accelerate gains from QI and prepare health systems to tackle uniquely African health challenges, among others. Available evidence from QI implementation in high-income countries simply will not transfer to most low-income settings.

With respect to MNH, the Network for Improving Quality of Care for Maternal, Newborn and Child Health has been established since 2017. Among its objectives are to strengthen national QI efforts and to share learning around capacity-building and implementation of QI between partner countries (World Health Organization, 2019). This paper attempts to support the effort by providing an example of how realist evaluation can be used to study QI implementation and generate transferrable programme theory that may optimize its implementation across contexts, with emphasis on health systems strengthening.

### Realist evaluation methodology

**Figure 1:**
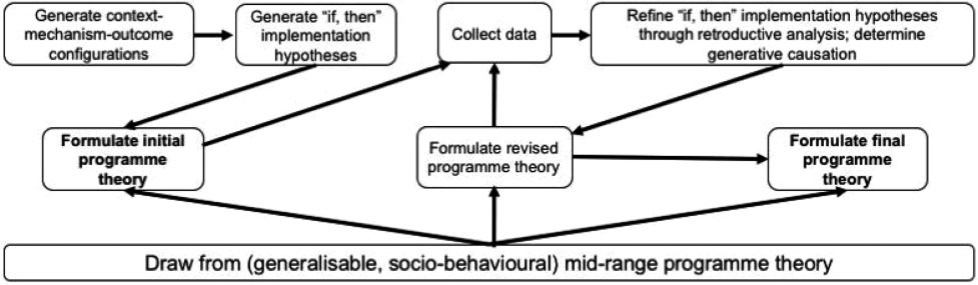
Realist evaluation lifecycle used to move from an initial to a final programme theory (adapted from Pawson and Tilley, 1997).

Realist evaluation is an implementation research approach. Implementation research is used to understand and overcome problems in implementing proven interventions. It is methods neutral, meaning that it can deploy a number of possible methods as required (World Health Organization and Special Programme for Research and Training in Tropical diseases, 2011). Realist evaluation is used to generate insights around implementation in the ‘real world’—specifically, what worked, for whom and under which conditions? It emphasizes the important role of context in moderating how an intervention’s inputs (e.g. training, resources provided) are responded to by recipients (called mechanisms), which produces specific outcomes. These can be explored at the individual, interpersonal, institutional and infrastructural levels (Pawson, 2002). These relationships can be described as context-mechanism-outcome configurations, and form the basis of implementation hypotheses, which are continuously refined throughout the life of an intervention. These implementation hypotheses are used to build an initial programme theory. This theory helps to identify what could *possibly* work, for whom and under which conditions. This, too, is refined throughout the life of a programme as field data around implementation are continuously collected and analysed, explaining, testing and revising implementation hypotheses until a ‘final’ programme theory for the intervention is produced, which describes what *did* work, for whom and under which conditions (Pawson and Tilley, 1997). Mid-range programme theories are broadly generalizable social theories within classes of interventions, and can be used to inform these realist programme theories. As such, in addition to providing excellent documentation and analysis of implementation, realist evaluation is hypotheses-testing and theory-building (Figure 1) (Pawson and Tilley, 2004).

### Using realist evaluation to study QI

The distinction between realist approaches and other evaluation methods is the emphasis on understanding causation. As pointed out by Fletcher et al.(2016, p. 288), ‘there is an inherent compatibility of complex systems science, critical realism and realist evaluation in their mutual commitment to understanding causality within complex environments’. Realist principles can, however, be integrated within existing research or evaluation methods, as well as being an evaluation method itself. Realist evaluation has been explored in conjunction with other methods, e.g. action research (Westhorp *et al.*, 2016) and grounded theory (Bunt, 2018). This brings us to the application of realist evaluation to QI within health systems, which are inherently complex. There are many examples where realist evaluation has been used to study the implementation of QI interventions in high-income countries—which is where the evidence base for QI is concentrated more generally see examples: Schierhout *et al.*, 2013; Balasubramanian *et al.*, 2015; McConnell *et al.*, 2015). However, despite an increase in the use of realist evaluation across different interventions, this has been little-applied to study QI in low-income countries, and never, to our knowledge, with a view to health systems strengthening and MNH.

QI can generate context-specific insights around health systems bottlenecks and ways to overcome these. Realist evaluation can provide needed insights around what worked, for whom and under which conditions in QI. Realist evaluation can also generate a programme theory, which has application outside of the setting of implementation. These insights may speak to key aspects of implementation, such as generating capacity to carry out the QI intervention, operationalizing the intervention and sustaining it in the absence of external facilitation from outside organizations.

Here we introduce an example of a realist evaluation to evaluate an ongoing complex, multi-level QI intervention in southern Tanzania, aimed at improving MNH outcomes. We present our initial programme theory for QI implementation, built using realist principles. We then introduce how insights gained from understanding mechanisms that underlie implementation can be harnessed to support better implementation and operationalization of QI for health systems strengthening.

## Materials and methods

### Intervention setting

The QI at District Scale for Improvement in Maternal and Newborn Health (QUADS) intervention takes place in four districts in Mtwara region in southeastern Tanzania: Newala (population 205 492), Tandahimba (population 227 514), Masasi District Council (population 247 993) and Masasi Town Council (population 102 696) (National Bureau of Statistics, 2013). This region has been historically disadvantaged and, until recently, has had among the poorest MNH indicators in the country (Ahearne, 2016). The population is predominantly Muslim and from the Makonde ethnic group. The primary economic activity here is farming (The Planning Commission United Republic of Tanzania, 1997). In Tanzania, the institutional delivery rate is constantly climbing, sitting now at 63%, however, the maternal mortality ratio and neonatal mortality rates have remained largely unchanged over the past 15 years, and remain high, at 524/100 000 live births and 25/1000 live births, respectively (National Bureau of Statistics and ICF Macro, 2016; Ogbo *et al.*, 2019; World Health Organization *et al.*, 2019). In Mtwara region, 99% of women attend antenatal care at least once, but markers of quality of antenatal care provision are markedly lower, with only 55% of women having their urine tested and only 52% receiving two doses of the tetanus toxoid vaccination, for example. Eighty-two per cent of births are attended by a skilled provider, the majority of which occur in public-sector health facilities (National Bureau of Statistics and ICF Macro, 2016). The National Road Map Strategic Plan to Improve Reproductive Maternal, Newborn, Child and Adolescent Health in Tanzania (2016–20) guides MNH nationally. This strategic document highlights the importance of quality care and QI, especially at the facility level. However, it also highlights the importance of community sensitization and mobilization (The Ministry of Health, Community, Development, Gender, Elderly and Children, 2016).

Across Tanzania, health services are decentralized to each district’s Council Health Management Team (CHMT). The CHMT oversees the management of and resource allocation to the health facilities in each district. These are, at the lowest level, dispensaries—some of which cannot support labour and childbirth, followed by health centres—many of which do not offer caesarean sections, and hospitals—which should be able to offer a full range of comprehensive emergency obstetric care services.

### The QI intervention

QUADS is a 5-year intervention (2015–20) funded by the International Development Research Centre. It is based heavily on a sister intervention, the Expanded Quality Management Using Information Power (EQUIP) intervention (2011–14), which was only implemented in one of the four QUADS districts, Tandahimba [see Hanson *et al.* (2014) and Waiswa *et al.* (2017) for more detail, including around district selection]. QUADS is a multi-level QI initiative that creates QI collaboratives at the community, health facility and district levels. ‘QI collaboratives’ bring together QI teams for peer learning, sharing of best practices and healthy competition (Nembhard, 2009), which is the foundation of our approach to QI, adapted from the Institute for Healthcare Improvement (Institute for Healthcare Improvement, 2003). This approach trains QI teams to use PDSA cycles: Teams identify key problems in their setting (i.e. their community, health facility or district) and carry out a root cause analysis to determine the causes of these. They then, based on their analysis, design a strategy to implement to address these (plan). They then implement their strategies (do) and collect data to determine if improvements resulted (study). QI teams themselves collect data linked to each topic they are working on. They plot data before and after implementing solutions to the key barriers linked to each topic to determine if changes have resulted, usually each month. These are then used to make ‘run charts’ (Perla *et al.*, 2011), which are annotated to highlight key influential contextual factors (e.g. a stockout of a medication necessary to fulfil the QI-generated solution). Based on the extent to which improvement did or did not occur, teams then choose to adopt their strategy, adapt it or abandon it to try something new (act).

QI teams in each district are created at three levels, supported through mentoring and coaching. Mentoring and coaching at all levels involve helping teams to identify any challenges they are facing using PDSA cycles, and to address those challenges. Mentors will visit QI teams and assess their progress. They may provide hands-on training to develop QI capacities. They play a role in motivating teams to remain active in QI, especially by recognizing their successes and showing encouragement. QI mentoring and coaching is guided by a checklist where comments on progress, key areas of difficulty within the team and so forth, can be made. These are shared with QUADS staff so that they can solicit extra support as needed.

At the *community* level, two QI volunteers were recruited by their community (i.e. community members suggest volunteers based on who they feel may be suitable for the role) and are mentored by a village leader with technical support from staff from their local health facility. Communities involved are from the catchment areas of health facilities that are part of QUADS. A Community Development Officer, who is employed by the district to facilitate development activities, also supports community QI teams, following up with QI volunteers and village leaders to assist in overcoming any challenges faced. At the *health facility level*, some or all of the maternity staff (and their supervisors) providing care to mothers and newborns, depending on the size of the facility and the number of staff, form a QI team. Facilities include one-third of each district’s dispensaries and health centres, and each district hospital. These teams are mentored by the district’s Reproductive and Child Health Coordinator, who already has supervisory responsibility for MNH services at participating facilities. At the *district level*, CHMT members make up the QI team. CHMT members are mentored by QUADS project staff [one community development specialist (EM) and one clinical specialist (MM)] directly, with support from the Regional Medical Office.

In addition to mentoring and coaching, a second key QUADS activity is learning sessions, which facilitate QI collaboratives. These occur once every 3–4 months. They bring together QI teams across each level (e.g. a community learning session, a health facility learning session and a CHMT learning session), though some participants from different levels are invited to attend other learning sessions (e.g. CHMT members are invited to health facility learning sessions). These introduce QI topics, agreed upon as target areas from the outset of the intervention due to poor coverage or performance, narrowed down by regional and district medical officers from the intervention area. We also aimed to align topics with routinely collected data, so that collecting additional data for QI activities would not be overly burdensome for participants. Topics include: early uptake of antenatal care (on or before 12 weeks’ gestation); four or more antenatal care visits; early uptake of postnatal care; improved quality of postnatal care; clean birth/infection prevention and control; active management of the third stage of labour (health facilities only); management of post-partum haemorrhage (health facilities only); and neonatal resuscitation (health facilities only). CHMTs work on resource allocation and management issues linked to these topics as relevant. Topic-specific training is provided at learning sessions if required.

To the extent possible, teams across levels work on different aspects of the same topic area in order to facilitate synergistic improvements. For example, completing four or more antenatal care visits was a topic introduced to both health facilities and communities, as it requires action at both levels to ensure success.

**Figure 2:**
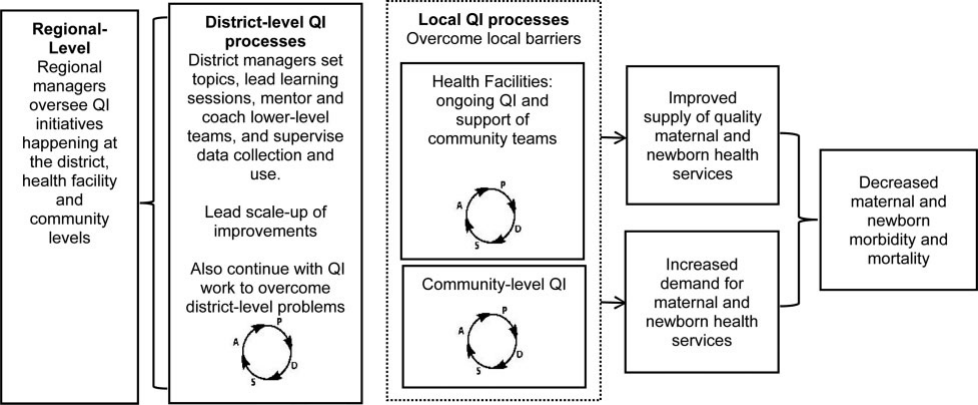
Conceptual model for district-led QI to reduce maternal and newborn morbidity and mortality.

External facilitation by QUADS project staff will be phased out to have mentoring and coaching and learning sessions run exclusively by the CHMTs in each district, with overarching leadership from the Regional Medical Office (Figure 2). As such, mentoring and coaching of health facilities will be done entirely by Reproductive and Child Health Coordinators in each district, and within communities, it will be done by village leaders in conjunction with local health facility staff, with overarching support from the district Community Development Officer. CHMT QI activities will be overseen by a chairperson with support from District Medical Officers. Furthermore, the most impactful solutions generated by QI teams will be summarized into ‘change packages’, with detailed suggestions for their implementation. These will be introduced across the district, led by the CHMTs, as a means of scaling up improvements. Overall, QI activities should improve MNH care and contribute to a reduction in preventable maternal and newborn morbidity and mortality.

#### Realist evaluation: generating an initial programme theory

Several steps were used to elicit the initial programme theory, aligned with realist methodology. First, we carried out a scoping review across relevant literature in order to identify candidate mid-range programme theories that may be applicable to our initial programme theory. This included searching databases (PubMed/MEDLINE, Global Health, PsychINFO, Web of Science, Google Scholar) with respect to theories underpinning QI interventions, team work, health management and community participation. Empowerment theory (see Results section) emerged as the most promising candidate mid-range theory.

Second, available data on implementation of other relevant QI interventions were collated. We reviewed publications from the EQUIP intervention (Tancred, 2016; Tancred *et al.*, 2017, 2018; Waiswa *et al.*, 2017; Baker *et al.*, 2018) (some of which were produced from extensive qualitative data from QI team members and mentors at the community, health facility and district levels) and other collaborative QI interventions with multi-level components in LMIC settings (Singh *et al.*, 2013; Cofie *et al.*, 2014; Tesfaye *et al.*, 2014; Kumar *et al.*, 2019). From the EQUIP intervention, the following additional documents were reviewed: learning session minutes, mentoring and coaching records, and EQUIP project staff reports. Macro and meso contextual data around socioeconomic factors, demographic factors, environmental characteristics, baseline health conditions, health service characteristics, and the presence of other QI programs were derived from reports from the Mtwara Commissioner’s Office, Comprehensive Council Health Plans for each district, census data, reports from the Medical Department Stores and also from annual key informant interviews with the Mtwara Commissioner, the Mtwara Regional Health Secretary and Regional Medical Officer and the Tandahimba District Health Secretary and District Medical Officer.

We analysed data retroductively, meaning that we worked backwards from patterns in outcomes (called demi-regularities) to try to explain *why* (called generative causation) they resulted (the mechanisms), what inputs from the intervention enabled the mechanisms to take place, and what contextual factors influenced both the mechanisms and the outcomes (Pawson and Tilley, 2004; Wong *et al.*, 2016; Gilmore *et al.*, 2019). The outcome patterns are therefore understood as existing within a broader system, and we aimed to understand what it was about the systems that created the outcomes we saw (Pawson and Tilley, 2004). We also used an elicitation research approach, in which many different sources of data (from QI teams, qualitative data and contextual data) were drawn together (Pawson and Tilley, 2004). From these, we generated a series of context-mechanism-outcome configurations. We then established a set of core ‘if-then’ implementation hypotheses generated from the context-mechanism-outcome configurations (see Table 1 in Results section) that draw on our candidate mid-range theory (Empowerment theory). Finally, an initial programme theory, based on these implementation hypotheses and empowerment theory was described in terms of health system building blocks.

**Table 1:** Initial context, mechanisms and outcomes anticipated for QUADS and resulting implementation hypotheses

Building block	Context	Mechanisms	Outcomes	Resulting implementation ‘if, then’ hypotheses
Service delivery	*Health facility:* Inconsistent quality in services	Through use of QI, capacities are built and teams become more adept at generating successful improvement strategies around targeted processes of MNH care, which reinforces teams’ interest in the use of QI	Better quality of key MNH services	Against the backdrop of poor quality of care, if teams consistently apply QI methods, then capacities will be built and teams will be more adept at generating successful improvements, which will positively impact on quality of care, reinforcing interest in the use of QI.
Health workforce	*All levels:* Participants are motivated by a genuine interest to improve MNH outcomes; participants lacking skills and confidence in QI *Health facility:* High staff turnover; heavy workload shared amongst small number of staff	Through use of QI, participants see positive changes, which reinforces their belief that they can make improvements for mothers and newborns, which reinvigorates their interest in using QI Learning session attendance will facilitate sharing of best practices due to the existence of common barriers and will further spark ‘healthy competition’ to overcome these, motivating teams to carry out QI with the recognition that it can be used with success, as demonstrated by other teams Learning session attendance and mentoring and coaching helps to maintain organizational QI memory by facilitating relevant clinical and QI skill development in new and current participants, thus instilling in participants the confidence that they can carry out QI. Carrying out QI will then further reinforce these skills and capacities.	Use of QI Better MNH outcomes Required clinical and QI skills and capacities present	If participants believe that they have the capacity to change maternal and newborn health outcomes through QUADS (i.e. are empowered), which is reinforced by seeing positive changes resulting through QI activities, then, given their genuine interest in improving MNH outcomes, QI participants across levels will be motivated to carry out QI activities and will use QI, continuously producing better MNH outcomes. If participants attend learning sessions, then they will engage in the sharing of best practices due to the existence of common barriers and will further participate in ‘healthy competition’ to overcome these, motivated by the recognition that QI can be used with success, as demonstrated by other teams. If participants attend learning sessions and receive regular mentoring and coaching, then they will develop requisite clinical and QI skills necessary to use QI—as many will not have any prior QI experience—thus building their confidence in their ability to actually carry out QI, resulting in more QI activities, which further reinforces skill development.
Health information	*All levels:* Inadequate data literacy and numeracy skills across some participants using QI (especially in districts where there have been no prior QI activities)	Learning sessions and mentoring and coaching will specifically target skill gaps (such as collecting, plotting, and analysing data), leading to the use of these skills, further reinforcing them	Required data literacy and skills in data use present Use of QI	If participants attend learning sessions and receive regular mentoring and coaching, then they will develop requisite data literacy and skills in data use necessary to use QI—as many will not have any prior QI experience—thus building their confidence in their ability to carry out QI, resulting in more QI activities, which further reinforces data literacy and skills in data use.
Medical products, vaccines and technologies	*Health facility:* Chronic undersupply of drugs and equipment needed to carry out strategies from QI effectively (e.g. oxytocin)	Liaison between levels at learning sessions provides a platform through which QI participants at lower levels may advocate for resources that can be made available by participants at higher levels, which would otherwise be constrained due to hierarchical social structures	Resource generation	Given inconsistent supply of necessary drugs and equipment, if lower-level QI participants (e.g. at health facilities) are able to discuss these constraints and advocate for resource generation with participants at higher levels (e.g. within the CHMT) through platforms created through QUADS (e.g. learning sessions), then participating health facilities will have better access to required drugs and equipment.
Financing	*Community:* Lack of resources at the household level, impeding families to act on care-seeking and good household-level care practices	Recognition—through local data collection as part of QI—the need for targeted strategies to assist vulnerable families, resulted in local resource- generation strategies	Creation of emergency transport funds to facilitate uptake of care	Given poor resources at a household level, constraining care-seeking and good household-level care practices, if QI is used, then the need to strategize around resource generation for the most vulnerable households will be recognized, and initiatives to create resources for these households, such as the establishment of emergency transport funds, will be enabled.
Leadership and governance	*Community:* External leadership from community mentors (supplied by the intervention); internal leadership from village leaders *CHMT:* Existence of national QI mandate; decentralised leadership to CHMTs *All levels:* No financial incentives for QUADS participation	*Community:* Given social structure, buy-in from village leaders will increase community acceptance of QI participants, facilitating access to households *CHMT:* Existence of QI responsibilities within district managers’ job descriptions/something that should be completed in their day-to-day supervision activities will also mean availability of some time and resources (e.g. access to a vehicle) that can be ‘piggybacked’ on to complete QI activities *All levels:* Regular mentoring and coaching will prompt team leadership and accountability, facilitating regular use of QI, resulting in improvements, will sustain QI participation	QI activities carried out QI skills built and sustained	Given the social structure of villages, if local leaders are involved in supporting QI activities, then that will facilitate community acceptance of QI activities led by local QI participants, and will further enable access to households where this may be necessary as part of QI strategies created through QUADS. If there is a QI mandate set within their job descriptions/something that should be completed in their day-to-day supervision activities, then district health managers will have time and resources to complete QI activities, and will, additionally, have a sense of responsibility to do so, and will be more likely to participate in QUADS. If there is regular mentoring and coaching, then it will facilitate good leadership and accountability among QI teams, prompting QI participation. Teams using QI will make improvements, thus being motivated to continue its use, as there are no direct financial incentives to carry out QI within QUADS.

This initial programme theory has become the basis of the field data collection and analysis that has taken place and that will take place throughout the lifespan of QUADS. This, and the resulting final programme theory, will be described in future publications.

## Bringing findings from QI and realist evaluation together for health systems strengthening

From realist evaluation, key implementation insights that can be influenced throughout QUADS implementation have been collated and summarized (see the black box in Figure 4). These have been generated from the mechanisms indicated in Table 1. Understanding generative causation and the relationship between context, mechanisms and outcomes, facilitated the creation of these implementation insights. The outcomes from Table 1 have also been summarized (see Table 2) with respect to the possible impact of well-implemented QI on health systems.

**Table 2:** How health systems building blocks may be strengthened through QUADS

Building block	How it may be strengthened through QI in QUADS
Service delivery	Improvements in service linked to specific improvement topics (e.g. content and uptake of postnatal care; active management of the third stage of labour; use of clean birthing practices)
Health workforce	Motivated and empowered staff; health facility staff better equipped with transferrable problem-solving skills
Health information	Improved data literacy and numeracy skills; better routine data entry (especially where these data are used in QI)
Medical products, vaccines and technologies	Procurement of required drugs and equipment through improvement strategies and advocating for resources at the district level; mobilizing resources after being alerted to gaps from the district-to-health facilities
Financing	Mobilization of funds as necessary through QI (e.g. establishing emergency transport funds)
Leadership and governance	District managers (i.e. CHMT members) better equipped with transferrable problem-solving skills; district managers trained to support QI

## Results

### Findings about QI implementation: generating an initial programme theory from realist evaluation

#### Candidate mid-range theory: empowerment theory

Empowerment has many definitions and here it is understood as a ‘process by which people gain control over their lives, democratic participation in the life of their community, and a critical understanding of their environment’ (Perkins and Zimmerman, 1995, p. 570). It is borrowed initially from industry, where empowerment was applied within the context of total quality management, which lauded bottom-up approaches to problem-solving, and ultimately ended up restructuring workplace hierarchies. Empowered employees could self-actualize and make contributions on the basis of their skills (Wilkinson, 1998). Empowerment theory has long since been embraced within healthcare and development and particularly, in community participation (Fawcett *et al.*, 1995). Empowerment theory links individual capacities to broader social well-being. Empowerment-orientated interventions are those that aim to problem-solve, and provide opportunities for those engaged to build and strengthen their capacities and to engage collaboratively with others—in this respect, empowerment-orientated interventions seek to overcome social hierarchies. Empowerment hinges on participation around shared goals and a shared understanding of the socio-political environment, action around which may facilitate access to resources (Rappaport, 1981; Perkins and Zimmerman, 1995).

In prior QI interventions, it was clear that participants seeing improvements as a result of their own action was empowering (Tancred, 2016; Tancred *et al.*, 2017, 2018). Many participants within EQUIP expressed that they were now in a position to make changes where they previously could not. Establishing platforms for liaison brought together participants who otherwise would not be, all with an equal opportunity to discuss issues, transcending in some ways the existing social hierarchies. Having an opportunity to advocate for needs at higher ‘levels’ within the health system contributed to the sense of empowerment felt by participants in EQUIP (Tancred *et al.*, 2017, 2018; Baker *et al.*, 2018), and we expect the same in QUADS.

Learning sessions provide a platform for sharing of best practices and encourage healthy competition. In EQUIP, sharing between teams enabled lower-performing teams to see that, even in facilities with similar problems and constraints, it was possible to make improvements. There were also consistent efforts made to celebrate improvements and acknowledge efforts, which was motivating and contributed to participants’ sense that they could achieve success through QI.

#### Context-mechanism-outcome configurations

**Figure 3:**
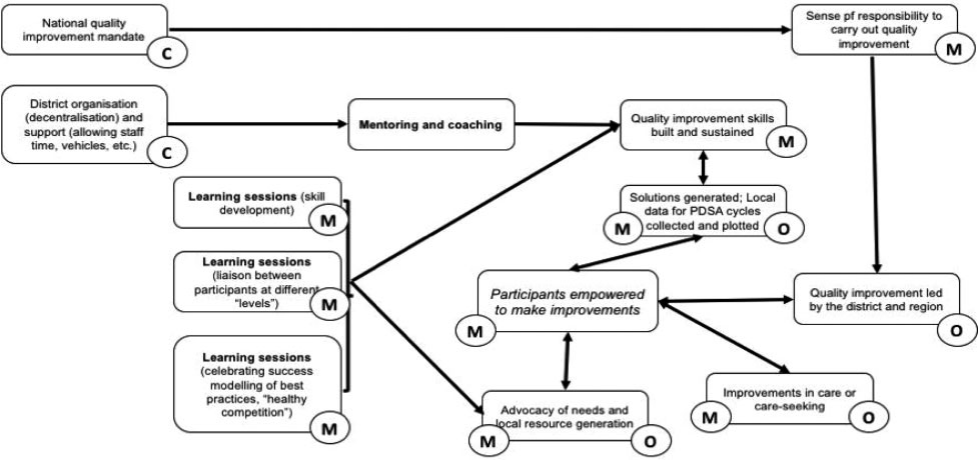
Visual representation of context, mechanisms and outcomes used to build ‘if, then’ hypotheses and the initial programme theory.

Following retroductive analysis and elicitation from across sources, Table 1 below highlights core context, mechanisms and outcomes anticipated in QUADS. This visual depiction of the initial programme theory (Figure 3) highlights the specific context (delineated with ‘C’), mechanisms (delineated with ‘M’) and outcomes (delineated with ‘O’) present. Programme/intervention inputs are bolded. Here, some outcomes are self-reinforcing of themselves or other outcomes, as indicated through the bidirectional arrows and designation as both a mechanism and an outcome. The central role of empowerment as a fundamental mechanism is highlighted in italics.

#### ‘If, then’ hypotheses

We reorganized context-mechanism-outcome configurations in the form of ‘if, then’ hypotheses around implementation, organized by health system building blocks. Where a specific level (community, health facility, CHMT or all) is implicated, this is highlighted in the Context column.

#### Initial programme theory

These implementation hypotheses, taken from a lens of health system strengthening and drawing from empowerment theory, enabled us to produce our *initial programme theory:* Within the context of decentralized district health management teams with a mandate to support QI, QI team members (the *health workforce*) who believed that it is possible to make improvements (i.e. empowered members) were more likely to participate, and therefore, were more likely to thoughtfully identify problems and solutions, collect and plot local data (*information*), tangibly see improvements (in *service delivery*, among others), and in doing so, reinforced their belief that change could take place, which was further empowering. Teams and individual team members were empowered through a facilitating environment provided through the intervention. This involved creating platforms for skill-building, liaison with other QI teams across ‘levels’—enabling advocacy of needs, including petitioning for supplies (*medical products*/*vaccines/technology*) and mobilizing local resources (*financing*)—celebrating success, modelling of best practices and fostering healthy competition between teams through learning sessions and mentoring and coaching (*leadership and governance*), which helped teams to overcome skill deficits and equipped them with the confidence to start and sustain QI activities.

### Bringing realist evaluation and QI together

**Figure 4:**
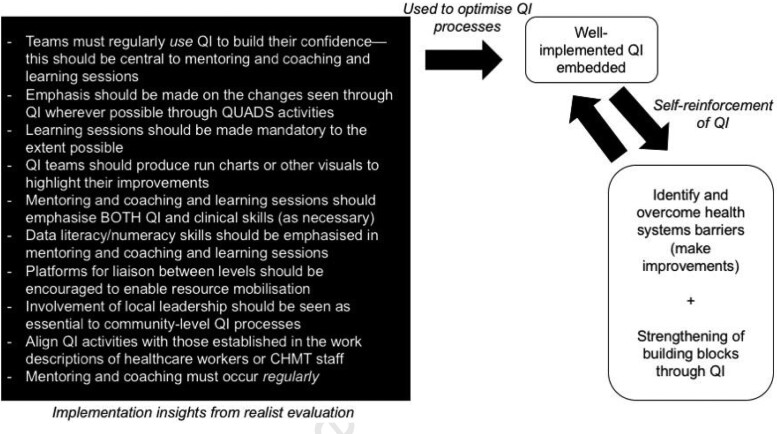
Demonstration of how realist evaluation and QI can be used, together, to strengthen health systems.

As identified through our realist evaluation, we have uncovered how QI may also strengthen health systems building blocks, summarized in Table 2. However, to achieve positive outcomes, QI must be well-implemented by practitioners with strong QI skills (e.g. good data literacy/numeracy, keen problem identification through root cause analysis, innovative but feasible strategy creation). Realist evaluation may be used to understand *how*, *for whom* and *under which conditions* outcomes linked to implementation are achieved (e.g. the ‘if, then’ hypotheses in Table 1). Therefore, it can identify what can be introduced through an intervention to best capitalize on this, given a holistic understanding of the complex system in which the QI takes place and the moderating role of context. Figure 4 provides a summary of these implementation insights in the black box, generated through the ‘if, then’ hypotheses from the realist evaluation. These can be used to optimise QI processes, thus resulting in embedded, well-conducted QI, leading to improvements in care provision and care-seeking and strengthening of health systems building blocks. As also learned from realist evaluation, these outcomes then further the conduct of well-implemented QI. The centrality of this empowering process has also been articulated in our initial programme theory which, following revision throughout the life of QUADS, can be useful in understanding how QI interventions in similar contexts may be expected to work.

## Discussion

### Bringing the two together: overcoming health system bottlenecks through optimizing QI implementation

Through the processes of QI, health systems building blocks may be strengthened. However, insights from realist evaluation are useful in appreciating how, for whom and under which conditions health systems building blocks may be strengthened. In turn, modifiable aspects of a QI intervention to optimize QI processes and to maximize health systems strengthening can be identified. Without the use of realist evaluation—e.g. if implementation had been studied using process evaluation—we would not have identified mechanisms that had been triggered through the QI intervention, and therefore, would be left without valuable insights about how to modify the intervention in order to trigger those mechanisms within the study context. We have demonstrated the transition from big mid-range programme theories (empowerment theory), to programme theory (our initial programme theory) to actionable implementation considerations through the ‘If, then’ hypotheses.

Furthermore, to the best of our knowledge, this is the first realist evaluation-generated initial programme theory for QI, produced with a view to health systems strengthening, generated from empirical and theoretical data from LMIC contexts. Implementation hypotheses will be explained and revised throughout the course of the QUADS realist evaluation. We aim to then produce a final programme theory at the conclusion of QUADS, which can be used to facilitate other health systems strengthening interventions through the use of QI, especially in LMICs. Without the use of realist evaluation, generating a robust programme theory would not be possible.

The insights from such research—specifically with a view to the unique study design of QUADS—responds directly to a call to strengthen implementation research in LMICs by ensuring that interventions occur ‘under usual management conditions, employ a pragmatic research paradigm, and address critical implementation issues such as scale-up and sustainability of evidence-informed interventions’ (Alonge *et al.*, 2019a, p. 2). Furthermore, a recent review and consultation defined a framework of core implementation research competency domains relevant to practitioners in LMIC settings. These include applying structured processes to identify bottlenecks in the health system (achieved through QI); identifying emerging challenges related to implementation of evidence-based interventions (achieved through QI with insights from realist evaluation); contextualising health system bottlenecks that constrain implementation of evidence-based interventions (achieved through QI and realist evaluation); stakeholder engagement (achieved through QI); conducting and monitoring implementation research (achieved through QI in terms of local data-driven monitoring of improvement, and broader evaluation of implementation and outcomes in realist evaluation); and feeding results into the health system (achieved through both QI and realist evaluation) (Alonge *et al.*, 2019b). As such, when brought together, researchers and practitioners can develop relevant implementation research competencies through the practice of QI and realist evaluation.

#### Methodological complementarity within implementation research

Future implementation research can build on the insights here and should aim to continue developing other methods to facilitate the robust study of implementation for knowledge translation (Straus *et al.*, 2009). The use of complementary implementation research methods should be considered. QI itself can be considered an implementation research approach, offering opportunities to learn about the operationalization of effective solutions to overcome health systems bottlenecks. With insights gained from realist evaluation, the two together: generate learning about the role of context; allow for in-depth exploration of implementation (including capacity-building, operationalization, scale-up and sustainability); emphasize learning about implementation within ‘real-world’ conditions, which facilitates knowledge translation; measure both implementation and outcomes, enabling insights around processes and impacts; generate transferrable programme theory through realist evaluation for otherwise context-specific QI implementation; and generate actionable implementation recommendations through realist evaluation to optimize QI processes.

Furthermore, the Standards for QI Reporting Excellence (SQUIRE) guidelines emphasize a need to report on context (nature and characteristics of the setting), detailed intervention description (including changes to implementation—inclusive of how and why plans evolved, success of implementation and so forth) as well as changes in care or patient outcomes (Ogrinc *et al.*, 2015). Realist evaluation facilitates well reporting around such elements, which are often under-reported in QI interventions.

#### Reflections on health systems strengthening through realist evaluation and QI

We may better understand the health systems gains from well-implemented QI identified through realist evaluation (more motivated staff, better quality care and care-seeking, better local data collection and analysis around healthcare and health-seeking processes, local resource generation, and better stewardship of problem-solving activities), which are relevant across the health system. There is therefore considerable potential for ‘diagonal’ health systems strengthening—generated through QI, and maximized through insights from realist evaluation—which is increasingly applied with success in LMICs (Gounder and Chaisson, 2012; Mahomed *et al.*, 2014; Knaul *et al.*, 2015; Orenstein and Seib, 2016).

#### Limitations

Both QI and realist evaluation require extensive amounts of data to be collected, collated and analysed by skilled individuals. Efforts to strengthen both QI and realist evaluation skills among LMIC researchers and practitioners would be of value, but will require dedicated funds, political commitment and technical support from local government and potentially also international organizations. Furthermore, as indicated in the introduction, there may be considerable barriers to QI implementation. However, realist evaluation may identify these and highlight platforms through which they may be overcome.

One key drawback of this approach is that, as implementation research, it does not *necessarily* involve rigorous impact evaluation, operating under the assumption that QI can work to improve health systems and health outcomes, and therefore, what is needed most is to understand how to implement and operationalize QI processes. This gap in high-quality impact evaluations of QI interventions, especially in LMIC settings, has been flagged by other researchers and practitioners (Garcia-Elorrio *et al.*, 2019). As such, future research that rethinks how QI is evaluated, rather than repeating poor-quality evaluations with limited robust, independent data collection and synthesis, would be of value, preferably alongside detailed study of implementation. Furthermore, if realist evaluation is not carried out in a timely fashion, with findings feeding into the implementation of QI, it may not actually support the optimization of QI processes. However, these findings may still be of use to the design of future QI interventions.

With respect to the application of realist evaluation, it is perhaps most meaningfully utilised by researchers/practitioners from the setting in which the intervention is taking place. There may be limitations when realist approaches are used by researchers external to a setting. These may include power imbalances which impact interviewing; key details being literally ‘lost in translation’, with a commensurate impact on drawing out the most relevant context-mechanism-outcome configurations; loss of insights around context due to a lack of contextual familiarity; and over-reliance on theories that are derived from ‘Western’ settings (Gilmore, 2019). Increased use of realist evaluation by LMIC researchers directly would be enormously beneficial.

To respond to Heiby’s call (2014), establishing Africa-based networks for sharing learning around QI for health systems strengthening would be of particular value. Current networks like the African Forum for *Quality Improvement* in Healthcare (International Society for Quality in Health Care, 2019) and, with a view to MNH, the Network for Improving Quality of Care for Maternal, Newborn and Child Health (World Health Organization, 2019), could be capitalized on and expanded to this end.

## Conclusion

Realist approaches can evaluate and support QI activities used to overcome health systems bottlenecks to produce rich data about how to best equip and support local QI teams, considering the moderating role of contextual factors. They can generate important understanding about *how* health systems building blocks may be strengthened through QI processes. Additionally, they produce transferrable, empirically generated theories to share this learning across contexts. Realist evaluation is a robust implementation research approach to assess QI, maximizing potential implementation insights that would be of value to local decision-makers, as well as decision-makers in contexts with similar constraints, for health systems strengthening.

## Funding

This work was funded by the International Development Research Centre (grant number: IMCHA108020). We would also like to express our sincere thanks to Chris Bonell, Brynne Gilmore and G.J. Melendez-Torres for their thoughtful reading and comments on drafts of this manuscript. This article is part of the supplement ‘Innovations in Implementation Research in Low- and Middle-Income Countries’, a collaboration of the Alliance for Health Policy and Systems Research and Health Policy and Planning. The supplement and this article were produced with financial support from the Alliance for Health Policy and Systems Research. The Alliance is able to conduct its work thanks to the commitment and support from a variety of funders. These include our long-term core contributors from national governments and international institutions, as well as designated funding for specific projects within our current priorities. For the full list of Alliance donors, please visit: https://www.who.int/alliance-hpsr/partners/en/.

*Conflict of interest statement.* None declared.

*Ethical approval.* Ethical clearance has been obtained from the research ethics committees of: Ifakara Health Institute, the National Institute for Health Research (Tanzania), and the London School of Hygiene and Tropical Medicine.
